# Medical Opinion and Movement

**Published:** 1908-11-14

**Authors:** 


### Medical Opinion and Movement.

An extremely important observation on the prophylaxis of syphilis by calomel ointment is reported in the New York *Medical Record* by Dr. Wolbarst. The author points out that the classical experiments of Metchnikoff upon Dr. Maisonneuve, though controlled as carefully as possible by simultaneous experiments on apes, are not proof-positive of the efficiency of the prophylactic action of this drug. They are open to several objections besides the one cardinal source of fallacy in the shape of individual immunity, which varies so markedly in regard to the disease. Dr. Wolbarst had, quite fortuitously, a chance of testing certain points, which were not cleared up by Metchnikoff, in the following way. He was consulted by a young man who, with a companion, had exposed himself to contagion from a very active lesion only a short space of time previously. The companion had already started by train for the other side of the continent, was therefore already beyond reach, and thus acted as a ready-made human control. The patient was treated, within six hours of the first exposure, by the Metchnikoff-Maisonneuve method, and entirely escaped infection. The companion developed undoubted syphilis. The patient from whom they had both accepted the risk was examined and displayed numerous acute secondary lesions. The case is so important as a confirmation of the previous work that it may be taken to be as near absolute proof as we are likely to get as to the causal relation of the inunction and the subsequent escape from infection. To put on record at once such a remarkable case was a clear duty, and the author is to be congratulated on the upshot of his treatment.

Adenitis as a complication of the administration of serum for diphtheria is described by Dr. J. D. Rolleston in the *British Journal of Children's Diseases*, occurring at the same time as rashes and other serum phenomena. It has often been observed that enlargement of the inguinal glands is common as an early sequela of serum injection, but it is the submaxillary and cervical groups which react later. The author has taken considerable pains to distinguish between this adenitis and that which does at times occur after diphtheria without serum treatment, and has satisfied himself that whereas the latter is found in only 2½ per cent. of cases, the serum reaction is present in more than 9 per cent. Also adenitis as a response to serum treatment occurs earlier than that which is simply a sequela of the disease. It is more frequent after severe attacks, probably because the adenoid tissue is then in condition of less resistance to inflammation. Complete resolution is the rule, and suppuration the exception; persisting chronic enlargement is quite uncommon. This serum adenitis has no prognostic significance, needs no treatment beyond fomentations to the neck unless suppuration occurs, and has to be diagnosed from a late adenitis due to the original diphtheria, from intercurrent tonsillitis, and from other acute exanthemata.

The production of new hypnotics and their introduction to the profession under various names has now been going on steadily for many years, and the supply shows no sign of slacking off. Each drug is, according to the advertisements of its vendor, the ideal hypnotic, efficient, harmless, and incapable of setting up a drug habit. Each in turn is the cause of fatalities and other disasters, reported in a minority of cases only in the columns of the medical Press. Particularly valuable, therefore, are the unbiassed investigations of Dr. Ollerenshaw into the therapeutics of some of the newest of these drugs, published in the *Medical Chronicle*. Thus chloretone is, he believes, of use for sea-sickness when there is already gastric disturbance, but of no avail against the usual type of cerebral or cerebellar origin. It has very feeble hypnotic powers, but has proved very efficacious in the treatment of chorea. Brometone has effects very similar to chloretone, but has rather more hypnotic action It was tried for epilepsy on three patients: one did very well, the other two did not. Chloralamide suffers under the great disadvantage of a long latent period; thus, if given overnight, it sometimes has no effect until the next day. It is only moderately efficient as a hypnotic, but does not seem to have any depressant results. Its taste is objectionable, but this is counterbalanced by the safety of the preparation. Phenalgin and ammonol are two proprietary preparations of very similar composition; both are mixtures which owe their main effect to acetanilide, which is generally regarded as the most dangerous of the coal-tar hypnotics. From practical experience the author declines to recommend phenalgin.

A more favourable verdict is passed upon veronal, whose scientific name is diethylbarbituric acid. Like chloralamide this drug is best given dissolved, to avoid the possibility of long delayed action; it is more soluble in hot water than in cold, and in alkalies than acids. Veronal has been known to produce fatal results even in 10-grain doses, but these or any other ill effects are extremely rare. Its chief merit seems to be that the sleep induced is more like natural sleep than that which follows the administration of any other hypnotic. Dr. Ollerenshaw thinks it does better for severe cases of insomnia than for those who are merely living at high pressure; for the latter he prefers bromural, which is not quite so strong as veronal. He has used it with marked success for the insomnia of the overworked examination candidate and for that of anæmia, and thinks highly of it. As far as efficiency goes he places isopral midway between these last two drugs. It acts very rapidly, but has a most unpleasant taste, which can be got over by administration as an inunction. If care is not taken the nurse who carries out the inunction may also become drowsy. Neuronal is more potent than any of the other compounds tested; it appears to have no prejudicial action on normal hearts, but is distinctly contra-indicated for insomnia due to cardiac disease, in which also it seems to have but little effect. The system rapidly becomes accustomed to the drug, which is of most service for mania and similar conditions, but mental depression follows its use too often for routine use as a hypnotic.

---

Moro, of Munich, who is one of the chief assistants of Professor Pfaundler at the local children's hospital, reports *in extenso* his experience of carrot soups in the treatment of gastro-intestinal disorders in children, to which we alluded in a note in these columns some months ago. The diet faddist has even entered the children's ward, and the general practitioner is chary of accepting as gospel everything that is claimed for some new foodstuff for children's use. Moro's conclusions, however, appear to be based on a series of instructive cases in which the treatment recommended has undoubtedly been of the greatest benefit. Acute as well as chronic cases of gastro-intestinal disease were treated, and in every one there was a most encouraging betterment to be observed soon after the treatment was begun. The vomiting, diarrhœa, and restlessness stopped; the patients became quiet and slept well. Usually they improved in weight, and a most striking feature is the quick fall in the temperature. It seems rather difficult to explain this latter, for the author does not claim for his carrot soup the action of a definite antipyretic. Its usefulness may lie in its cellulose contents, which stimulate the intestinal mucosa. Probably the sugar which the soup holds in solution is more rapidly absorbed under those conditions, but on this point it is impossible to speak definitely. Moro remarks that patients on such a diet improve very rapidly provided there is no marked carbohydrate intolerance. The author has found that when the soup is prescribed there soon appears a change in the stools. They lose their foul smell and fewer Gram-positive organisms are to be found in them. The treatment is now being given a prolonged trial at various children's hospitals on the Continent, and the author promises a further and more extensive report on it at an early date.

---

Rimbaud reports a case of post-influenzal polyneuritis. This complication is by no means rare, and a history of recent influenza, or some febrile catarrhal condition which it is excusable to look upon as influenzal, is sometimes obtained in cases of multiple neuritis. Rimbaud himself is inclined to believe that the influenza bacillus is the direct cause of the nerve lesion, but the evidence he brings forward to support such a conclusion is slight. His patient, a shepherd aged 57 years, was, previous to his illness, an exceptionally healthy man who had never had syphilis, and who very rarely took alcohol in any form. The patient was attacked by a subacute type of influenza, from which he made what was apparently an excellent recovery. The temperature sank to normal, and he got up and appeared to be rapidly recovering. At this stage the typical symptoms of a multiple neuritis developed. A feature of the case was the excessive pain of which the patient complained when he was handled. The superficial reflexes were abolished in the lower limbs: in the arms the signs were not so well marked. Urinary and rectal troubles complicated the case. Under electrical treatment the patient gradually, but very slowly, improved, and finally completely recovered. Rimbaud, in view of the fact that the ordinary causes of acute polyneuritis could be more or less definitely excluded in this case, diagnosed a typical post-influenzal multiple neuritis, and points out that in some ways the case presented features which likened it to the ascending type described by Landry. The prognosis in the influenzal type is, however, distinctly good; but, as in this case, recovery is very slow. Internal medication seems useless, and the mainstay of the treatment must be in electrical stimulation.

---

The frequent cure that follows a simple exploratory laparotomy in cases of tuberculous peritonitis is already well established, and it has been supposed that the value of the treatment lies in the admission of air into the peritoneal cavity through the abdominal incision. Acting upon this supposition the suggestion presents itself that the introduction of oxygen into the abdominal cavity might be of still greater efficacy. Dr. MacGlinn reports in the *New York Medical Journal* upon four cases of tuberculous peritonitis of the fibrous type in which he claims to have obtained a complete cure by this method of treatment. A median abdominal incision is made, and through this sterilised oxygen is passed into the peritoneal cavity from a special sterilised apparatus till the abdomen is distended. The incision is then closed and the gas is left in contact with the peritoneum for some minutes. It is then allowed to escape again and the operation is repeated. In this way the abdomen is charged and recharged with oxygen for the space of about 30 minutes, and this has proved sufficient in each case to effect a complete cure. In one case the cure dates back as far as four years ago.

---

In a paper read before the Académie des Sciences, M. Répin seeks to establish a relation between the goitre-inducing properties of the waters of certain districts and their radio-activity. He has found three sources of the Maurienne, notably "goitregenous," to be also radio-active owing to emanations of radium, and he has extended his researches to other sources found to be radio-active from other substances, especially thorium. He examined 14 springs in Savoy and was able to detect by means of an electroscope the emanations from water flowing in sheets for several hours. After a descent sometimes of 2,000 metres the waters were still radio-active. He cites a case of particular interest at Bourg d'Oisans, where he found a well, the water of which was radio-active, and of four people who drew from the well three already had goitre and the fourth was developing it. The disease was quite unknown in the town itself, where the water supply was from the river. The author concludes, therefore, that the "goitregenous" waters of the Alps are especially radio-active and that this radio-activity is due for the most part to radio-thorium. It seems possible, therefore, that radio-activity is a common attribute of all water rising from a great depth and coming into contact with volcanic rocks, and may explain the association of goitre with mountainous districts.

---

An interesting case of arterio-venous anastomosis is recorded by Wieting, of Constantinople, in the *Deutsche Medizinische Wochenschrift*. Ordinary measures of treatment for gangrene of the extremities resulting from arterio-sclerosis are so unsatisfactory and so often lead to amputation as a last resort, that the idea occurred to Wieting that the arterial circulation might be carried on through the veins, as these vessels are usually much less affected by the disease. A patient, aged forty, had had the right leg amputated in 1906 for gangrene and this year the left leg developed in its turn the same disease. The author exposed the femoral artery high up close to the deep femoral. He compressed the artery by means of catgut against a cylinder of wood covered with caoutchouc. The artery was then divided and the peripheral end ligatured. The compression remained on the central end. The femoral vein was then ligatured and compressed in the same way as the artery. On the anterior surface of the vein and on the peripheral side of the ligature a small incision was then made, into which the central end of the femoral artery was introduced and stitched by circular sutures. The two vessels were gradually relieved from the compression and the wound sutured. The result was completely satisfactory. Two months afterwards the foot was quite warm and of normal colour, without any morbid symptom. The effect was immediately apparent on the next day after the operation.

---

The method of estimating the cardiac sufficiency or power of compensation as conceived by Katzenstein, is little known in this country. According to this authority, if a large artery (the external iliac for instance) be compressed, the blood pressure is raised throughout the whole cardio-arterial system. A normal heart should be able to respond to the consequent increase of work without any increase in the frequency of the pulse. Katzenstein has practised this compression on invalids, while making at the same time observations on the arterial tension and pressure and the pulse rate, and distinguishes four classes, according to the amount of increase in the blood pressure and the presence or absence of increase in the pulse rate. In normal individuals, and in those even with hypertrophy of the left ventricle, there is sufficient reserve of cardiac energy to meet the call, but in cases of cardiac insufficiency, hyposystole, or failure of compensation, the pulse rate is increased and there may be actually a fall in blood pressure. Bonardi has recently made similar observations on one hundred patients of all varieties of maladies, and confirms the results of Katzenstein. He finds that convalescents from infectious diseases of all kinds belong to the fourth class, that is to say, they show a diminution of blood pressure and increase in the pulse rate. Bonardi makes a further distinction in regard to the duration of these changes. In cases of slight cardiac weakness they may last only a few minutes, but in the more severe form some hours. In cases of neurasthenia cardiac exhaustion can easily be demonstrated in this way.

Huchard and Fiessinger, in the *Journal des Practiciens*, discuss the treatment of cardiac arhythmia. They classify arhythmias into two main varieties—(1) functional; (2) organic. Functional arhythmia may arise from excitation of the central nervous system, and this excitation may be spontaneous or the result of some toxic condition. The former may follow fatigue or violent emotion; coffee, tea, or alcohol may cause the toxic variety. Rest and change of surroundings, with tepid douches, usually suffice to cure the spontaneous functional arhythmia; removal of the cause will cure the toxic variety. The authors are of opinion that when digitalis is given in functional cases it should be ordered in small doses, especially when it renders the pulse intermittent; they consider 5 minims of a one per cent. solution daily for a fortnight suitable treatment. This should be followed by an interval of about a week before the drug is ordered again. Functional arhythmia is often associated with digestive disturbances, in which case the authors have used bismuth subnitrate with success. This is given about an hour before meals, and continued for a fortnight. Then cachets of equal parts of bicarbonate of soda, prepared chalk, and magnesium hydrate will be found of benefit. The patient should drink little during a meal, but some infusion, such as camomile tea, may be given an hour after meals.

---

Organic arhythmia, according to the authors, should be treated with ice-packs to the heart, and in jections of strychnine. Digitalis should only be given in small doses. In chronic cases 5 minims of a one per cent. solution of digitalin may be given, and liquids reduced from the diet. If there be arterial degeneration the diet should be confined to milk and vegetables, while theobromine may be given in addition to digitalis. This will cure dyspnœa even if the arhythmia persists. In obese patients a reduction of fat will often cure the condition without medication. When there is old myocarditis the authors prefer strophanthus to digitalis, and use theobromine as well. In cases of mitral stenosis with arhythmia, digitalis and theobromine should be used alternately with liquor trinitrini. Where there is aortic insufficiency and a nervous temperament, subcutaneous injections of glycero-phosphate of sodium and valerian by the mouth are indicated. In Stokes-Adams disease atropine is useless, whereas in other forms of slow pulse-rate it accelerates the pulse in about half an hour. Slowing of the pulse due to partial suppression of the ventricular systole should be treated by removing the cause, *e.g.* auto-intoxication of intestinal origin.

